# Mélanome hépatique primitif: à propos d’un cas

**DOI:** 10.11604/pamj.2021.40.24.29557

**Published:** 2021-09-09

**Authors:** Mariama Jarti, Sara Boulajaad, Martial Ulrich Gouton, Adil Ait Errami, Zouhour Samlani, Sofia Oubaha, Khadija Krati

**Affiliations:** 1 Service de Gastro-enterologie, Centre Hospitalier Universitaire Mohammed VI, Marrakech, Maroc,; 2 Laboratoire de Physiologie, Faculté de Médecine et de Pharmacie, Université Cadi Ayyad, Marrakech, Maroc

**Keywords:** Mélanome, foie, primitif, à propos d’un cas, Melanoma, liver, primary, case report

## Abstract

Le mélanome malin est une maladie à fort potentiel métastatique qui se développe aux dépens des mélanocytes. Le foie est l´organe le plus souvent concerné par les métastases. Néanmoins le mélanome hépatique primitif est très rare. Peu de cas de mélanomes hépatiques primitifs ont été décrits. Nous rapportons le cas d'une patiente atteinte de mélanome hépatique primitif qui a été diagnostiquée par ponction biopsie hépatique, confirmé histologiquement et immuno-histochimiquement, avec une évaluation complète qui a permis d´exclure les autres mélanomes primitifs.

## Abstract (en)

Malignant melanoma is a disease with high metastatic potential which starts in melanocytes. The liver is the organ most often targeted for metastatic spread. Nonetheless, primary liver melanoma is very rare. Few cases have been described. We here report the case of a female patient with primary liver melanoma diagnosed based on liver biopsy results and confirmed by histological and immunohistochemical analyses as well as by providing a comprehensive assessment excluding other localizations.

## Introduction

Le mélanome est une tumeur maligne qui se développe principalement à la jonction de l'épiderme et du derme, ou dans des zones riches en mélanocytes, comme la peau, les muqueuses, le corps ciliaire des globes oculaires, l'iris, les choroïdes et les méninges. Bien que le foie soit un site fréquent de métastases des mélanomes, le mélanome hépatique primitif est très rare, et seulement quelques cas ont été rapportés dans la littérature [1]. Le diagnostic du mélanome hépatique primitif est difficile en raison de ses caractéristiques cliniques et radiologiques non spécifiques [2]. Nous rapportons ici l´observation d'une patiente atteinte de mélanome hépatique primitif qui a été confirmé histologiquement. L'objectif de ce travail est de rapporter les caractéristiques cliniques, radiologiques et thérapeutiques pour améliorer la compréhension du mélanome hépatique primitif et fournir un exemple pouvant servir de référence.

## Patient et observation

**Information de la patiente:** il s´agit d´une patiente âgée de 54 ans, sans antécédents pathologiques particulières, adressée dans notre unité pour la prise en charge des douleurs de l´hypochondre droit atypique, de siège fixe sans irradiation, sans position antalgique, d´intensité variable, évoluant de manière intermittente depuis 6 mois avant son admission. Aucune notion d´intoxication éthylique et aucune prise médicamenteuse n´étaient mentionnées.

**Résultats cliniques:** l´examen clinique était strictement normal, hormis une pâleur cutanéo-muqueuse et un certain degré d´asthénie avec une sensibilité de l´hypochondre droit en absence de signe clinique d´insuffisance hépatocellulaire. Les constantes hémodynamiques étaient correctes.

**Démarche diagnostique:** une numération formule sanguine de routine a donné les résultats suivants: numération leucocytaire = 11149/µL, neutrophiles = 69,5%, hémoglobine = 13 g/dL, les plaquettes étaient à 308 000/mm^3^. Le bilan biologique hépatique était le suivant: bilirubinémie totale à 5,9 µmol/L; activités sériques de l´ASAT est à la normale (N), de l´ALAT normale, de la GGT à 4N et des phosphatases alcalines à 2N. Le taux de prothrombine était à 70%. Les bilans sérologiques non contributifs: les anticorps anti-virus de l´hépatite C les IgM anti-HBc et l´antigène HBs étaient absents. Le scanner abdominal montrait un foie de taille normale et de contours réguliers siège au niveau du segment III et VI mesurant respectivement 5,1 x 4,1cm et 2,8 x 2,3cm spontanément hypodense se rehaussant discrètement après injection du produit de contraste. Des adénopathies sous hépatique mesurant 35 x 31mm, ovalaire hypodense au contraste spontané se rehaussant modérément après injection du produit de contraste (PDC). Absence de dilatation des voies biliaires intra et extra hépatiques. Les reins, les surrénales, la rate et le pancréas sont normaux. Un complément par IRM hépatique pour une meilleure caractérisation de la masse montrait un foie de taille normale de contours réguliers, siège de deux lésions nodulaires au niveau des segments III et VI du foie en isosignal TL, hyposignal T2 et diffusion avec restriction, avec composante kystique centrale en hyposignal T1, hyper signal T2, rehaussées de façon modérée et hétérogène sans *wash out* aux temps portal et tardif, mesurant respectivement 5,5 x 4,15cm et 3 x 2,6 cm. Absence de dilatation des voies biliaires intra et extra hépatiques. Il s'y associe une formation ovalaire sous hépatique sous-hépatique mesurant 3,5 x 3,1cm, en iso- signal hétérogène T1, hypersignal T2, renfermant des zones kystques, réhaussée de façon hétérogène par le contraste avec restriction de la diffusion au niveau des portions charnues.

**Figure 1:**
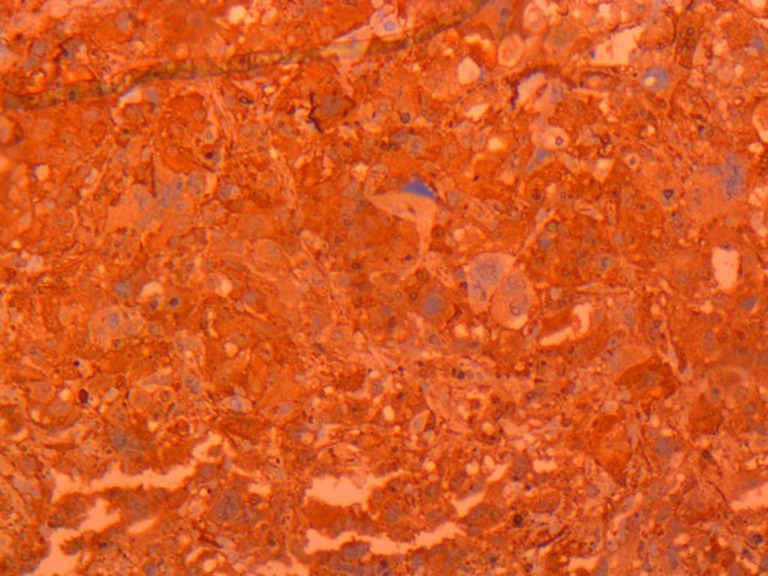
marquage cytoplasmique fortement positif aux anticorps anti HMB 45

**Figure 2:**
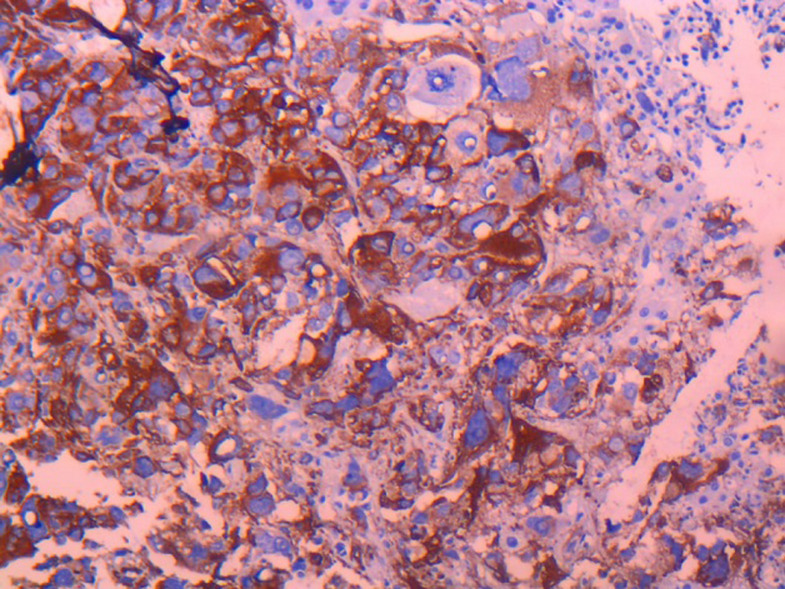
marquage cytoplasmique et nucléaire fortement positif aux anticorps anti PS 100

Une ponction biopsie hépatique à l´aiguille pratiquée par voie trans-pariétale révélait un fragment hépatique. L´étude histologique du fragment hépatique montrait une localisation hépatique d'une prolifération tumorale maligne à grandes cellules géantes parfois multi nucléées. Ces cellules renferment par endroits un pigment brunâtre. Le cytoplasme est abondant. L´étude immunohistochimique sur coupes en paraffine montrait la présence de l'anti-mélanosome HMB45 (clone HMB45 HexaBiogen) et l'anti-protéine S-100 (15e2E2+4C4.p Blocare) était positive et l'anti-hépatocyte (clone OCH1E5 Hexa Biogen) était négatif (Figure 1, Figure 2). La recherche d´un mélanome primitif extra-hépatique était négative. Des consultations dermatologiques et ophtalmologiques également réalisées, n´ont pas mis en évidence un mélanome. Une fibroscopie avec un examen proctologique ne révélant pas d´anomalie notable. Le diagnostic de mélanome hépatique primitif est finalement retenu. Un bilan d'extension a été réalisé et il comportait une tomodensitométrie thoraco-abdomino-pelvienne à la recherche d'une extension locorégionale et à distance mettant en évidence un nodule pulmonaire d'allure suspect avec une lésion ostéolytique mitée per trochantérien gauche sans réaction periostée ni atteinte des parties molles en regard.

**Intervention thérapeutique et suivi:** une chimiothérapie à base de dacarbazine et immunothérapie par interféron ont été proposées. Mais la patiente a été perdue de vue après l'annonce du diagnostic.

## Discussion

Les mélanomes constituent 1 à 3% de la totalité des cancers [3]. Le plus souvent, ils sont cutanés ou développés aux dépens de la choroïde. Le risque de récidive et de métastase du mélanome malin est élevé et le pronostic est généralement mauvais [4]. Le primitif d´un mélanome métastasique est généralement identifié ; cependant, chez 3,2% des mélanomes métastatiques le primitif reste inconnu [2, 5]. L´étiopathogénie du mélanome hépatique primitif n'est pas connue : la régression (du primitif) suite à la propre réponse immunologique du patient est l'hypothèse la plus soutenue. Il n'y a pas de mélanocytes dans le foie. Quelques auteurs ont suggéré que ces néoplasmes résultent de mélanocytes ectopiques qui ont subi une transformation maligne [6, 7]. Ou bien une transformation maligne des cellules mélanoblastiques de la crête neurale au cours de l'embryogenèse [2]. Le diagnostic de mélanome hépatique primitif doit actuellement inclure les trois indicateurs suivants: mélanome hépatique confirmé histologiquement et immunohistochimiquement. L´exclusion d'autres mélanomes malins primaires, l'absence d'une tumeur cutanée antérieure (qui a été détruite ou excisée sans examen histologique) [1]. La symptomatologie clinique est variée et non spécifique, dominée par les douleurs abdominales, une masse abdominale et l´altération de l´état général [8].

La tomodensitométrie est la méthode la plus couramment utilisée pour diagnostiquer les lésions tumorales hépatiques, elle est cruciale pour la localisation et la caractérisation des tumeurs hépatiques [1]. L´étude de Weiquin et Ji propose les caractéristiques suivantes du mélanome hépatique primitif pour aider au diagnostic: lésions uniques ou multiples solido-kystiques occupant n´importe quel segment dans le foie, croissance expansive, une capsule est possible, saignement facile, nécrose centrale et lésion kystique; la tumeur peut présenter un rehaussement en forme d'anneau de fleur avec une zone de nécrose kystique centrale ne prend pas le contraste. L´imagerie par résonnance magnétique (IRM) est l´examen de choix, mettant en évidence une lésion spontanément hyper intense en pondération T1, rehaussée intensément par le gadolinium, et dont le signal s´atténue en pondération T2, orientant d´emblée le diagnostic vers un caractère mélanique ou hémorragique. De plus, ces résultats d'imagerie sont les plus caractéristiques du mélanome hépatique primitif [8].

Du fait de la rareté des cas rapportés, il n´existe pas de traitement bien codifié. En cas de lésion localisée, la chirurgie doit être proposée en première intention. Des études suggérèrent que la chirurgie avec curage ganglionaire dans le ligament hepatoduodenal peuvent être le meilleur choix pour les patients atteints de MHP résécable [1]. L´immunothérapie pourrait constituer une voie thérapeutique potentielle. L´interféron pourrait avoir un effet sur la survie sans récidive [9]. De fortes doses d'IL-2 ont suggéré par le *National Comprehensive Cancer Réseau* comme traitement de choix pour le mélanome avancé. La chimiothérapie (dacarbazine) a montré une efficacité limitée [10]. Cependant, plusieurs nouvelles molécules ciblées des thérapies telles que nivolumab et ipilimumab ont montré des résultats encourageants [11, 12]. La rareté de cette localisation explique l´absence de schéma thérapeutique validé.

## Conclusion

Les mélanomes primitifs hépatiques sont rares et leur caractère primitif est souvent difficile à établir. Pour retenir le diagnostic, il faut pouvoir, affirmer le caractère mélanique par l'histologie et l'immunohistochimie, éliminer tout mélanome primitif extra hépatique par un examen attentif de la peau et des muqueuses et enfin s'assurer de l'absence d'antécédent d'exérèse de lésions cutanées. La prise en charge thérapeutique n´est pas codifiée. Le pronostic est mauvais et associé à un taux élevé de récidives et une courte survie.
